# Methodological challenges and analytic opportunities for modeling and interpreting Big Healthcare Data

**DOI:** 10.1186/s13742-016-0117-6

**Published:** 2016-02-25

**Authors:** Ivo D. Dinov

**Affiliations:** Statistics Online Computational Resource (SOCR), Health Behavior and Biological Sciences, Michigan Institute for Data Science, University of Michigan, 426 N. Ingalls, Ann Arbor, MI 49109 USA

**Keywords:** Big data, Analytics, Modeling, Information technology, Cloud services, Processing, Visualization, Workflows

## Abstract

Managing, processing and understanding big healthcare data is challenging, costly and demanding. Without a robust fundamental theory for representation, analysis and inference, a roadmap for uniform handling and analyzing of such complex data remains elusive. In this article, we outline various big data challenges, opportunities, modeling methods and software techniques for blending complex healthcare data, advanced analytic tools, and distributed scientific computing. Using imaging, genetic and healthcare data we provide examples of processing heterogeneous datasets using distributed cloud services, automated and semi-automated classification techniques, and open-science protocols. Despite substantial advances, new innovative technologies need to be developed that enhance, scale and optimize the management and processing of large, complex and heterogeneous data. Stakeholder investments in data acquisition, research and development, computational infrastructure and education will be critical to realize the huge potential of big data, to reap the expected information benefits and to build lasting knowledge assets. Multi-faceted proprietary, open-source, and community developments will be essential to enable broad, reliable, sustainable and efficient data-driven discovery and analytics. Big data will affect every sector of the economy and their hallmark will be ‘team science’.

## Background

This article outlines some of the known barriers, intellectual and computational challenges, and opportunities in the area of big healthcare data (BHD). A blend of ‘team science’, open-source developments, engagement of diverse communities, innovative education and hands-on training will be essential to advance the field of biomedical research [1]. Technical problems, substantial resource costs, and the intellectual demands of handling, processing and interrogating BHD are barriers to advancement and progress. At present, a canonical framework for representation, analysis and inference that is based on incongruent, multi-source and multi-scale biomedical data does not exist. After two decades of rapid computational advances, a tsunami of data and substantial scientific discoveries, urgent unmet needs remain for (near) real-time predictive data analytics, (semi) automated decision support systems and scalable technologies for extracting valuable information, deriving actionable knowledge and realizing the huge potential of BHD.

The pillars of complexity science in healthcare include the diversity of health-related ailments (disorders) and their co-morbidities, the heterogeneity of treatments and outcomes and the subtle intricacies of study designs, analytical methods and approaches for collecting, processing and interpreting healthcare data [2]. In general, BHD has complementary dimensions - large size, disparate sources, multiple scales, incongruences, incompleteness and complexity [3]. No universal protocol currently exists to model, compare or benchmark the performance of various data analysis strategies. BHD sizes can vary, although complexity studies frequently involve hundreds to thousands of individuals, structured and unstructured data elements, and metadata whose volume can be in the ‘mega-giga-tera’ byte range. Such data often arise from multiple sources and can have many different scales, which makes modeling difficult. Finally, the complexity of the data formats, representations, sampling incongruences and observation missingness further complicates the data analysis protocols [4].

There are four phases in the analysis of BHD. The first phase is always to recognize the complexity of the process and understand the structure of the observed data as its proxy. Next comes the representation of BHD that should accommodate effective data management and computational processing. The last two phases of BHD analytics involve data modeling (including embedding biomedical constraints) and inference or interpretation of the results.

Innovative scientific techniques, predictive models and analytics need to be developed to interrogate BHD and gain insight about patterns, trends, connections and associations in the data. Owing to the unique characteristics of BHD, studies relying on large and heterogeneous data trade off the importance of traditional hypothesis-driven inference and statistical significance with computational efficiency, protocol complexity and methodological validity.

## Strategies, techniques and resources

### Structured and unstructured BHD

**Fig. 1:**
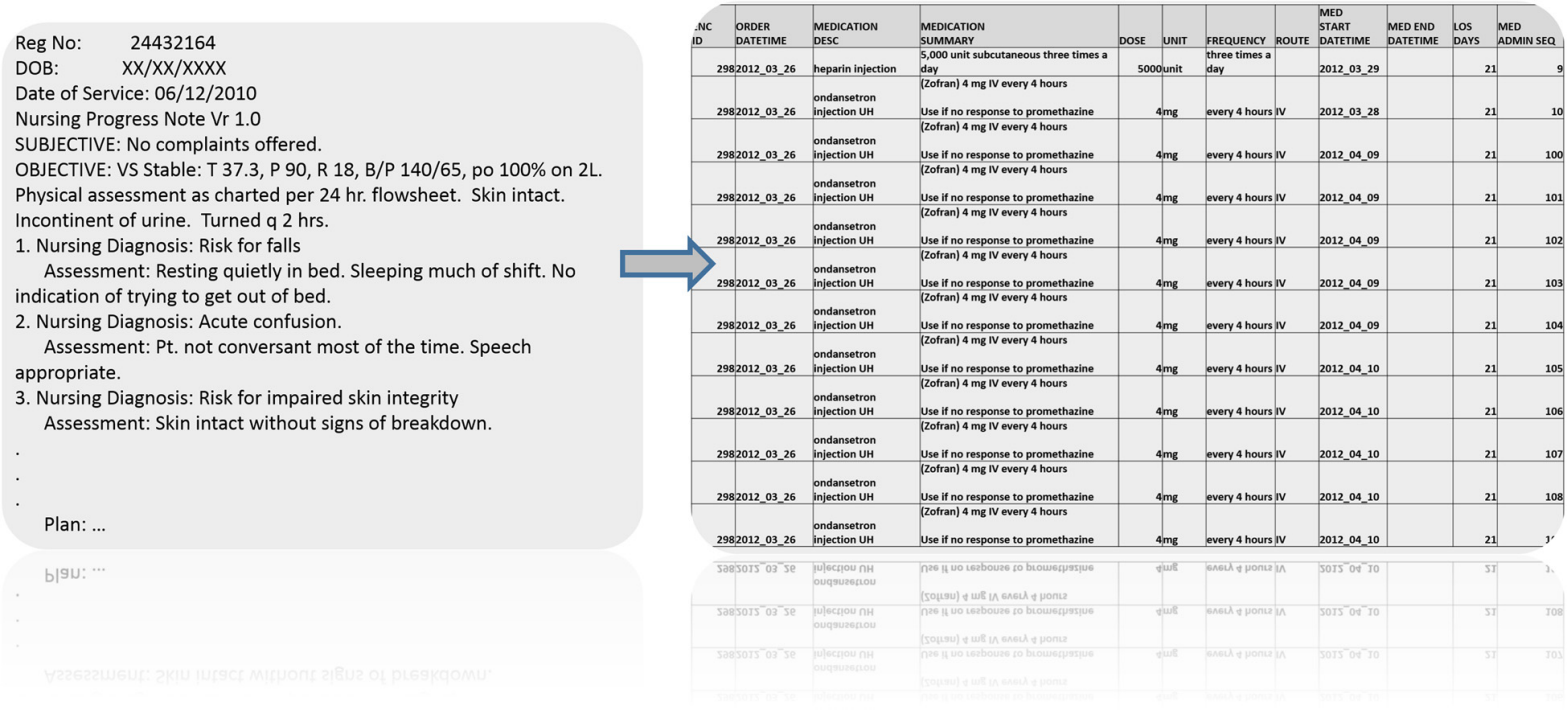
An example of parsing and interpreting unstructured clinical notes (left) and deriving structured data elements (right)

A key component of the complexity of BHD is the fact that most of the data is often unstructured, which means that in their raw format they are mostly qualitative or incongruent; this lack of congruence effectively stifles the ability to computationally process BHD [5, 6]. Examples of such unstructured data include raw text (such as clinical notes), images, video, volumetric data, biomedical shape observations, whole-genome sequences, pathology reports, biospecimen data, etc. Text mining [7], image or sequence analysis [8] and other preprocessing techniques [9, 10] need to be used to give structure to this unstructured raw data, extract important information or generate quantitative signature vectors. For example, text preprocessing can use statistical parsing [11], computational linguistics [12, 13] and machine learning [14] to derive meaningful numerical summaries. Information extraction approaches, such as entity recognition [15], relation extraction [16], and term frequency and inverse document frequency techniques [17, 18], provide mechanisms to extract structured information from unstructured text. Figure 1 shows an example of text parsing and semantic interpretation of clinical notes to obtain structured data elements that enable subsequent quantitative processing and statistical inference.

In the past decade, a sustained effort has been made to develop data standards, controlled vocabularies and ontologies for structural or semantic representations of data and metadata [19–22]. Specific examples of successful representation platforms for biomedical and healthcare data include minimum information standards. Examples of such standards include minimum information for biological and biomedical investigations (MIBBI) [23], minimum information about a microarray experiment (MIAME) [24], minimum information requested in the annotation of biochemical models (MIRIAM) [25], and core information for metabolomics reporting (CIMR) [26]. Examples of effective solutions and data standards developed and supported by various consortia include investigation/study/assay (ISA) [27], Clinical Data Interchange Standards Consortium (CDISC) [28], proteomics mass spectrometric data format (mzML) [29], and the nuclear magnetic resonance spectroscopy for metabolomics data markup language (nmrML) [27]. Powerful controlled vocabularies enable annotation, integration and servicing of millions of names, concepts and meta-data (e.g. diseases, conditions, phenotypes), and their relationships, in dozens of biomedical vocabularies, such as medical subject headings (MeSH) [30], gene ontology (GO) [31], and systematized nomenclature of medicine-clinical terms (SNOMED CT) [32]. Finally, there is a broad spectrum of domain-specific biomedical modeling standards, such as predictive model markup language (PMML) [33], XML format for encoding biophysically based systems of ordinary differential equations (CellML) [34], systems biology markup language (SBML) [35, 36], neural open markup language (NeuroML) [37] and tumor markup language for computational cancer modeling (TumorML) [38]. These architectures enable mathematical modeling and representation of biological constraints, and also promote machine-learning applications through the use of meta-learning schemes, data mining, boosting or bagging [39]. In a similar way, imaging, volumetric and shape-based observations can be preprocessed (e.g. by application of inhomogeneity correction [40], surface modeling [41], feature segmentation [42], etc.) to generate simpler biomedical morphometry measures, or biomarkers, that can be used as proxies of the raw unstructured data [43–46]. In general, summarizing data involves extractive or abstractive approaches for attaining structured information that is computationally tractable. Natural language processing (NLP) [47] is commonly used in healthcare, finance, marketing and social research as an abstractive summarization or a classification technique. Audio analytics (e.g. large-vocabulary continuous speech recognition) [48, 49] provide a mechanism for preprocessing and analyzing unstructured speech or sound data to facilitate subsequent extraction of structured information. Similarly, video content analysis (VCA) [50] can be used to monitor, analyze and extract summary information from live or archived video streams. In addition, such video analytics provide a valuable tool for longitudinal surveying, monitoring and tracking objects in 3D scenes.

### Graph networks

Social media applications, biomedical and environmental sensors, and municipal and government services provide enormous volumes of data that can carry valuable information. However, the informational content of such data might be hidden from plain view, entangled or encoded, which obfuscates the extraction of structured data and their interpretation in the networking context in which they were acquired. Content-based social analytics [51] focus on user-provided data in diverse social media platforms, wearables, apps and web services. Social data are always voluminous, unstructured, noisy, dynamic, incomplete and often inconsistent. In addition to the rudimentary challenges of managing such complex data, researchers encounter problems related to continuous software updates, technological advances (e.g. wearables), web server patches and product feature changes occurring during social studies.

**Fig. 2:**
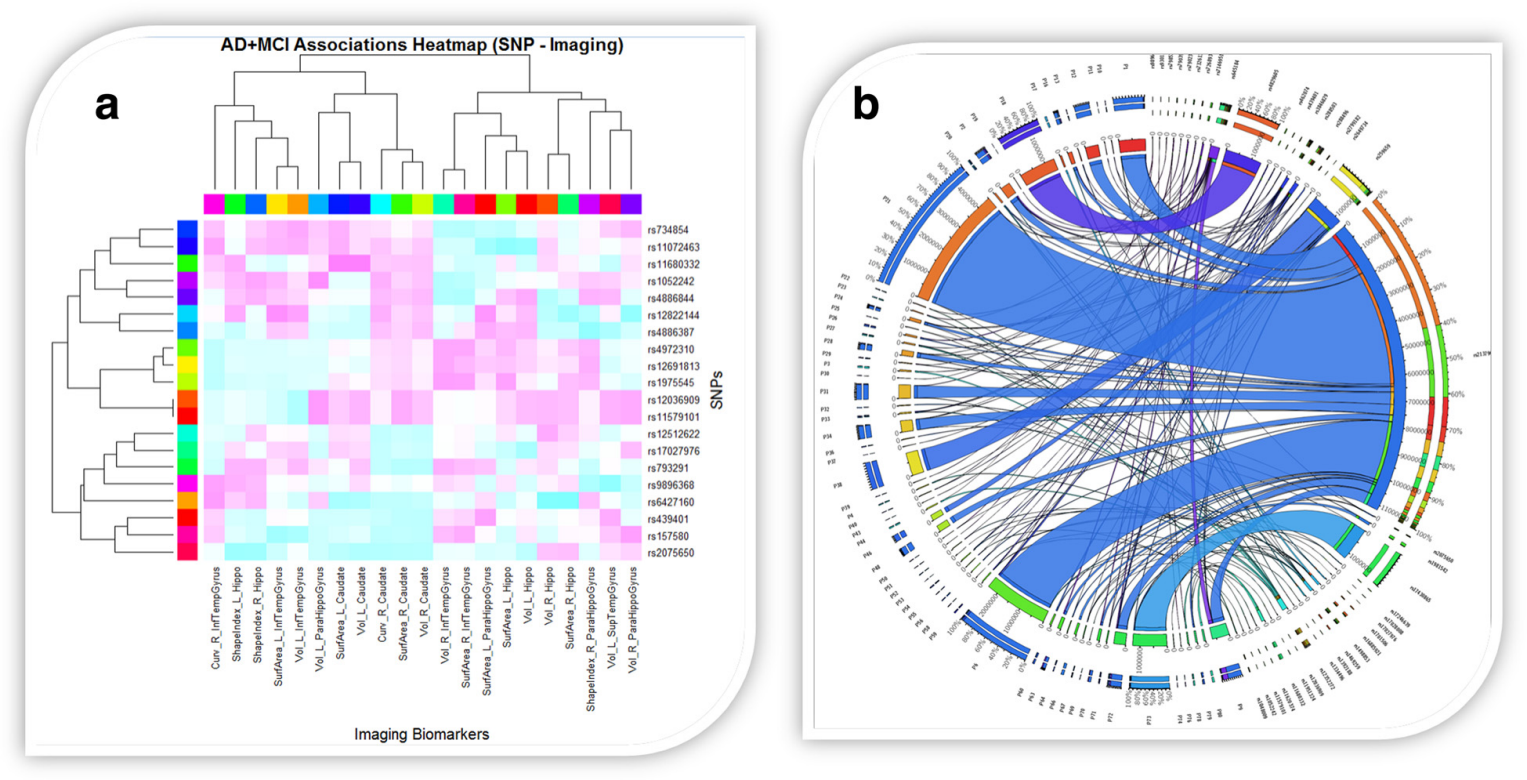
Examples of rendering complex network data. **a** A heatmap of neuroimaging-derived measures associated with individual phenotypes and genotypes [193]. **b** A Circos connectogram showing the associations (types and strengths) between genomics (single nucleotide polymorphisms) and neuroimaging (morphometry measures of brain regions) biomarkers [204]

Social network analytics [52] aim to harmonize, aggregate and synthesize structural attributes by using automated (unsupervised) [53] or semi-supervised algorithms [54] for data processing, discovery of relationships, or pattern extraction [55] among the participating social data entities. Social network modeling represents the data as a set of nodes (observations) and edges (relations between observations) that reflect the study participants and the associations within the network. Activity networks are a type of social graphs in which the nodes are either data elements or cases (participants) and the edges represent the actual interactions between pairs of nodes. Examples of interactions include dependencies (causal or relational) in which active relationships might be directly relevant to the network analysis. Social graphs are an alternative in which edges connecting pairs of nodes only signify the existence of a loose connection or weak link between the corresponding entities. Social graphs are useful to identify communities, clusters, cohorts or hubs. In scale-rich graphs, the connections between the nodes are uniformly random. Whereas in scale-free networks, the distribution of degrees of connectedness follows a power law with the increase in the number of nodes. Several powerful graphing methods exist for rendering, interrogating and visualizing complex network data [56–59]. Two network visualization examples are shown in Fig. 2.

Community discovery graph methods [60, 61] facilitate the implicit extraction of harmonious subgraphs within a network. Similar to clustering, community detection provides the means to summarize large networks, uncover intrinsic patterns or behaviors and predict critical properties of the network [62, 63]. Graph-based data mining can be used to partition networks into disjointed subgraphs (sub-networks, or hubs) on the basis of node similarity or distance measures. To model, evaluate and understand the influence of various nodes (actors) or edges (relations) in a social network we can use social influence analysis [64, 65]. As actions and behaviors of individuals within a social network affect others to varying degrees, assessing the joint influence of all participants on the entire community provides quantitative information about the strength of the network connections [66]. Social influence analysis captures the importance of nodes in the network and the stability, dynamics and efficiency of the entire social biosphere, and enables the modeling of influence diffusion through the network. Examples of specific approaches include linear threshold modeling and independent cascade modeling [67]. Various quantitative measures describing the social network characteristics can be defined [68]. Examples include measures of centrality (e.g. degree, betweenness, closeness, eigenvector or Katz centrality), graph distance measures (e.g. graph distance matrix, vertex eccentricity, graph radius), transitivity (e.g. graph reciprocity, global clustering coefficient, mean clustering coefficient), similarity (e.g. mean neighbor degree, mean degree connectivity, vertex dice similarity), etc. [69–71].

An important problem in social network research is predicting prospective linkages between the existing nodes in the graph network [72, 73]. The structure of social networks is mostly dynamic and continuously morphs with the creation of new or destruction and modification of existing nodes or edges. Understanding the internal network organization might enable the prediction of the dynamics or evolution of the network. Naturally observed networks, such as the internet, social networks, air-transportation networks and metabolomics networks, frequently share similar structural properties [74]. They are scale-free (with the fraction of network nodes with $k$ connections to other nodes following asymptotically a power law, *P*(*k*) ~ *k*^− *γ*^, for large *k*, with a power parameter typically $2<\gamma <3$) [75], and exhibit small-world features (all nodes, even non-neighbors, can be reached from every other node through a short sequence of steps. The six degrees of separation theory suggests that a chain of friendships between people can be made to connect any two humans in a maximum of six connections [76]. For example, network link prediction aims to estimate the chance of an interaction between entities and assess the influence among nodes in the network at a prospective time point [72]. Link prediction can also be used to examine associations in networks and to develop network decision support systems [77]. Network medicine is another example of a successful graph theoretic application [78], which uses functional interdependencies between cellular and molecular components to examine disease networks in situations in which several genes, multiple intracellular interactions and various tissue and/or organ systems jointly explain human pathology. Such networks enable the systematic exploration of molecular, environmental and genetic complexity for specific disease pathways and phenotypes.

### Classification

A plethora of algorithms, techniques and software tools are available for automated or semi-automated segmentation, clustering and classification of complex data [79–81]. Unsupervised machine-learning methods can be used to uncover patterns (or item sets) in numeric or categorical multivariate data [82, 83]. Bayes belief networks enable prediction, classification and imputation of missing values, and can be used to generate network representations of conditional dependencies among a large number of variables [84]. Deep learning is useful for complex unlabeled datasets and encapsulates machine-learning algorithms for organizing the data hierarchically and exposing the most important features, characteristics and explanatory variables as high-level graph nodes [85]. Ensemble methods combine the results from many different algorithms that vote in concert to generate increasingly accurate estimates. Compared with the results of any single algorithm or technique across the space of all possible datasets, ensemble methods provide highly effective predictive outputs [86]. Single-class classifiers are based on logistic regression and enable us to assess whether a data point belongs to a particular class. These classifiers can be useful in studies involving multiple cohorts in which the research interest is in identifying only one of many possible outcomes [87–89].

**Fig. 3:**
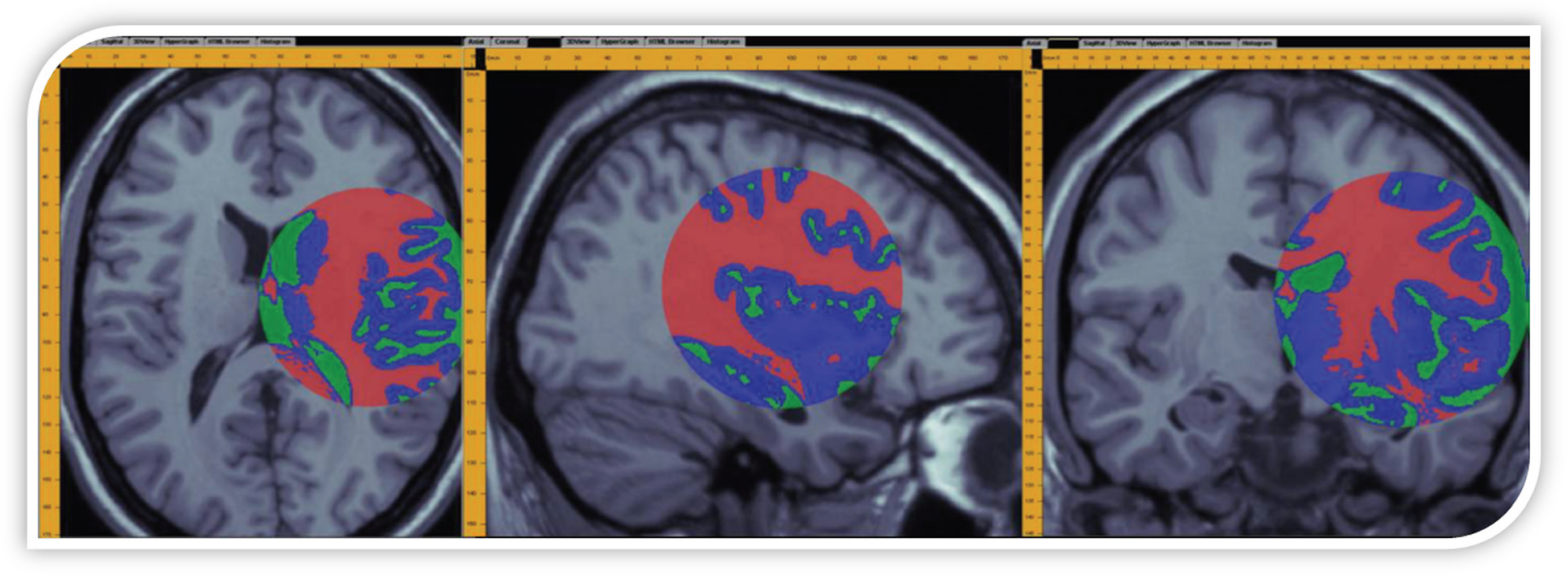
Example of using expectation maximization and Gaussian mixture modeling to classify stereotactic neuroimaging data [91]

Gaussian mixture modeling (GMM) represents an unsupervised learning technique for data clustering that uses expectation maximization to generate a linear mixture of clusters of the full dataset on the basis of univariate Gaussian (normal) distribution models for each cluster [90, 91]. Fig. 3 illustrates an example of using GMM to dynamically segment a 3D structural brain volume image into white matter, gray matter and cerebrospinal fluid. GMM algorithms typically output sets of cluster attributes (means, variances and centroids) for each cluster that enable us to quantify the differences and similarities between different cohorts. Random forests represent a family of decision-tree classification methods that produce a ‘forest of trees’ representing alternative models by iteratively randomizing one input variable at a time and learning whether the randomization process actually produces a more or less accurate classification result [92]. When the results are less or more optimal, compared to the results of the previous iteration(s), the variable is either removed from, or included into, the model at the next iteration, respectively.

*K*-nearest neighbors (kNN) classification algorithms [93–95] include the *K*-means methods for data clustering [96] and *K*-itemsets techniques [97] for association mining. These iterative methods partition a given dataset into a fixed user-specified number of clusters, *K*, which can be used to identify outliers as well as index, search, or catalog high-dimensional data. The local linear embedding method [98] is an example of a manifold learning method that aims to discover real, yet low-dimensional, topological shapes or patterns in the data [99]. Globally, the Euclidian representations of such shape manifolds can be warped and twisted. However, their intrinsic metric is locally homeomorphic to a lower-dimensional Euclidean distance measure [100]. For instance, consider the embedding in 3D of the 2D manifold representing the cortical surface of the human brain [101]. Cortical activation can be difficult to examine in 3D (because of the topology of the cortical surface); however, using the 2D manifold coordinates we can represent activation as data attributes anchored at vertices on the cortical surface. Another example is 3D data that live on a complex 2D hyperplane representing the linear associations of three variables representing the three natural base coordinates of the data [102, 103].

The different machine-learning (or statistical-learning) methods [104] are divided into supervised approaches (in which the goal is to use a training set that includes already classified data to draw inference or classify prospective, testing, data) [105] and unsupervised approaches (whose main task is to identify structure, such as clusters, in unlabeled data) [106]. Semi-supervised learning-based classification methods attempt to balance performance and precision using small sets of labeled or annotated data and a much larger unlabeled data collection [107]. Support vector machines (SVM) are powerful supervised machine-learning techniques for data classification [108] that use binary linear classification. SVM partition data vectors into classes on the basis of *a priori* features of the training data. SVM operate by constructing an optimal hyperplane (i.e. a maximum-margin hyperplane in a transformed feature vector space) that divides the high-dimensional dataset into two subspaces to maximize the separation of the clusters (for example, normal versus pathological cases). Boosting machine-learning methods create highly accurate prediction rules by combining many weak and inaccurate rules, associations or affinities detected in a (large) dataset [14, 109]. Adaptive boosting is one example in which the algorithm iteratively exploits misclassified examples from previous learning iterations and assigns them higher weights in the next round, which explains the adaptive influence, or iterative re-weighting, that is the signature feature of this method [110].

As the complexity of machine-learning algorithms can increase exponentially with the volume of the data, alternative model-based techniques, like generalized linear models (GLMs), may be more appropriate as they are computationally efficient and applicable for classifying extremely large datasets, [111, 112]. Using parallel processing [113], bootstrap sampling [114] and algorithm optimization [112, 115] can substantially improve the efficiency of all machine-leaning methods [116]. Compared with learning-based classification methods, such as SVM and boosting, the efficiency of GLMs in analyzing big data is rooted in their more simplistic linear modeling and regression estimation that make use of observed explanatory variables to predict the corresponding outcome response variable(s).

Examples of unsupervised quantitative data exploration and data mining algorithms for unlabeled datasets include association mining [117], link analysis [118], principal or independent component analyses (PCA/ICA) [119, 120] and outlier detection [102]. PCA projects high-dimensional data into a subspace of reduced dimension spanned by a family of orthonormal principal component vectors that maximize the residual variance not already present in the previous components. In practice, mutual orthogonality of the principal components might be a too strong assumption. Additionally, PCA relies on second-order statistics to estimate the covariances between the observed variables, which implies that the features that are generated might only be sensitive to second-order effects. Correlation-based learning algorithms such as PCA are designed to account for the amplitude spectra of data but largely ignore their phase spectra. This might limit their ability to characterize datasets with informative features that are modeled by higher-order statistics (e.g. skewness, kurtosis, etc.). ICA provides linear models for non-Gaussian data by generating components that are statistically independent. ICA model representations use blind source separation to capture the core structure of the data, which facilitates feature extraction and cohort separation. ICA is computationally efficient and applicable for data mining problems involving recovering statistically independent features from data assumed to represent unknown linear mixtures of attributes. Association mining represents another class of machine-learning algorithms applicable to large categorical data. This approach is mostly focused on discovering frequently occurring coherent associations among a collection of variables and aims to identify such associations on the basis of their frequencies of co-occurrence relative to random sampling of all possibilities. Link analysis aims to assign class labels to data elements on the basis of various link characteristics derived from iterative classification, relaxation labeling or other methods. Using link-based distance measures between entries we can generate associations expressing relative quantitative assessments of the between-element link associations in the entire dataset, extrapolate these patterns as network links, deduce novel plausible links and mine the collection. Many outliner detection methods exist for quantitative or qualitative detection of measurement errors, atypical observations, abnormal values or critical events [121].

### Incompleteness

Missing data arise in most complex data-driven inquiries [122]. To handle incomplete data, knowledge about the cause of missingness is critical [123]. If data are missing completely at random (MCAR), the probability of an observation being missing is the same for all entities [124]. In these situations, throwing out cases with missing data does not bias the final scientific inference. However, if the pattern of data missingness is not completely at random, such as when non-response rates are different in different subpopulations, the probability of observing an entity might be variable and we need to model, impute or correct for the missing values to obtain unbiased inference. We can model the process of missingness via logistic regression, in which the outcome variable equals 1 for observed cases or 0 for unobserved entities. When an outcome variable is missing at random (MAR), we can still exclude the missing cases as unobserved; however, the regression model should control for all the variables that affect the probability of missingness (e.g. object characteristics or subject demographics) [125]. Another common cause for incomplete data is missingness that depends on some specific unobserved predictors. Missingness not at random (MNAR) suggests that the incompleteness of the data depends on information that is not available, i.e., unobserved information may predict the missing values [126]. For instance, an aggressive cancer intervention can have side effects that make patients more likely to discontinue the treatment. Side effects and ‘discomfort’ associated with an intervention can be difficult to measure, which can lead to incomplete data due to MNAR. In such cases, we have to explicitly model the incompleteness of the data to avoid inferential bias. In certain situations, missingness can depend on the unobserved entity itself, that is, the probability of missingness depends on the missing variable [127]. For example, if younger adults are less likely to enroll in healthcare plans, case censoring may be in effect due to aging and we must account for the related missing-data by including more predictors in the missing-data model – that is, bring the process of missingness closer to MAR.

### Exploratory data analytics

Countless examples show the equivalence of a ‘word’ to a ‘thousand pictures’ [128] and its pseudo-converse that equates a ‘picture’ to a ‘thousand words’ [129]. Protocols for image parsing to text description (I2T) generate text from still images (or video streams) [130]. Conversely, exploratory data analytics transform text (tables) into figures (images) that represent a synthesized view of the information contained in the ASCII data. This duality of representation of complex information is also directly demonstrated by the homology between time-space and frequency (Fourier) representations of multidimensional data [131, 132]. Visual exploratory and explanatory analytics are critical components of any study of complex data. Such tools facilitate the graphical ‘storytelling’ of the properties and characteristics leading to, or explaining, BHD discoveries.

Data profiling is a collection of exploratory data analytic methods that facilitates quick and effective identification of some basic data characteristics [133]. Profiling evaluates the information content, intrinsic structure and quality of the data and explores variable relationships within them. Examining frequency distributions of different data elements provides insight into the type, center, spread and shape of each variable. Cross-variable analysis can also expose embedded value dependencies and discover overlapping or correlated features among the entities. Motion charts [134] are an interactive mechanism for mapping variables to different graphical widgets, which facilitates the dynamic traversal (playing the chart) across a time dimension. Typically, motion charts facilitate on-the-fly transformation of quantitative and qualitative information contained in multivariate data to expose relevant and actionable knowledge about the interplays among multiple data elements. ManyEyes data visualization [135] enables users to generate graphical displays of their own data. Socrata [136] enables the servicing and sharing of dynamic data via a user-friendly and cost-effective interface. D3 is a modern JavaScript platform for developing dynamic data visualizations. The Cytoscape visualization suite [56] enables exploration of network and tabular data. Several dashboard platforms exist (e.g. Tableau [137], SOCR MotionCharts [134] and SOCR Dashboard [138]) for interrogation of complex, structured or unstructured multi-source data. Data Wrangler [139] includes mechanisms for manipulating, transforming, filtering and visualizing incongruent data.

### Choosing the right statistical methodology

In terms of selecting appropriate statistical tests, the most important question is: ‘What are the main study hypotheses and specific goals?’ In some cases no *a priori* testable hypothesis exists; the investigator just wants to ‘see what is there’. For example, in a study investigating the prevalence of a disease, there is no hypothesis to test, and the size of the study is determined by how accurately the investigator wants to determine prevalence. If no hypothesis exists, then no corresponding statistical test are conducted. It is important to decide *a priori* which hypotheses are confirmatory (that is, whether we are testing some presupposed relationship), and which are exploratory (whether they are suggested by the data). No single study can support a whole series of hypotheses. There are a number of strategies to determine the most appropriate statistical tests and often alternative approaches need to be investigated. As there is no unique, complete, and consistent ontological hierarchy to guide practitioners, consultations with experts are useful. An example of a table of frequently used study designs and appropriate corresponding statistical analysis approaches is available online [140].

### Predictive analytics

**Fig. 4:**
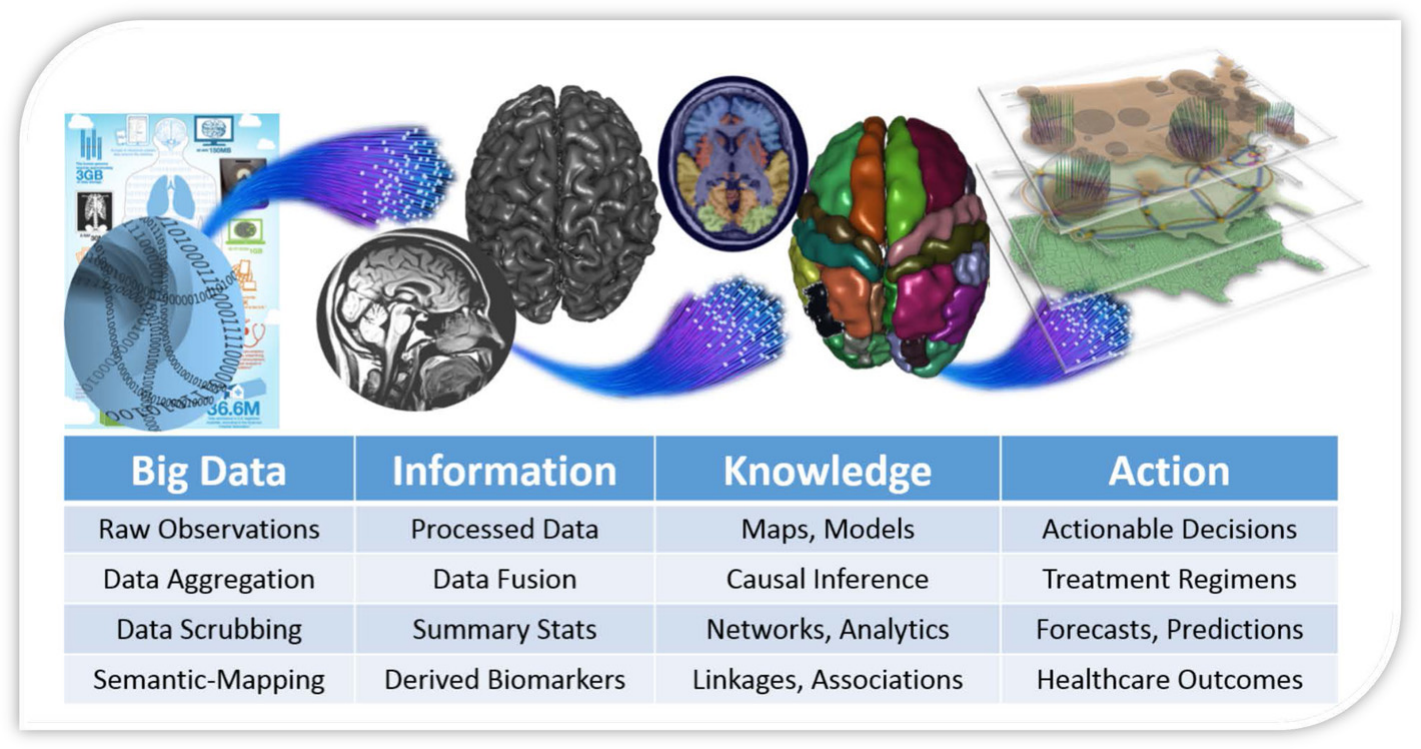
A schematic illustrating the big healthcare data analytic pipeline in a neuroscientific context, including data management, mapping, processing, interpretation and inference [138]

Large and complex clinical datasets require data-specific and study-specific analytic protocols for managing raw data, extracting valuable information, transforming the information to knowledge, and enabling clinical decision-making and action that are evidence-based (Fig. 4) [138]. Various methods exist to predict future outcomes or forecast trends using retrospective and current data. Predictive analytics are useful in all scientific inquiries or research explorations. Anticipating future failures or systemic changes using multi-source data streams that generate hundreds or thousands of data points is critical in decision-making, whether when buying a stock, preparing for natural disasters, forecasting pandemics, projecting the course of normal or pathological aging or anticipating the behavior of social groups. Predictive analytics aim to uncover patterns and expose critical relations in phenomena using the associations between data elements detected in the observed process. Two generic types of predictive analytics techniques exist: model-based or model-free. Predictive time series analyses can use moving averages to build a model using historical or training data and extrapolate the trend predicted by the model into the future. Multivariate regression methods [141, 142] represent variable interdependencies between predictors and responses in terms of some base functions (e.g. polynomials) whose coefficients capture the influence of all variables on the outcomes and facilitate forward predictions. Alternatively, machine-learning techniques [143], classification theory [144] and network analytics [145, 146] can be used for model-free (semi) unsupervised data mining, hierarchical clustering [147], pattern recognition [148], fuzzy clustering [149] or trend identification [150]. The type of outcome variables affects the analytical techniques used to study the process. For example, multilinear regression [151] is applicable for analyzing continuous outcome variables, whereas random forest classification [92] and logistic regression [152] can be applied to analyze discrete outcome variables.

Contemporary data science and analytic research demand innovative predictive forecasting and statistical methods that are capable of dealing with the complexity of big data that are prevalent in biomedical studies [153, 154]. Classical statistical methods are based on conventional complete data and specific *a priori* statistical significance assumptions. Scientific inference often depends on small data samples from a specific population with some assumptions on their distribution. To examine the significance of a particular relationship, statistical results are typically contrasted against random chance. Finally, data-driven findings might be generalized as a conclusion applied to the entire (unobserved) population. There are substantial differences in the sample attributes of traditional studies and big data studies. The latter are characterized by incompleteness, incongruency, multi-source elements, multiple scales, excessive heterogeneity, and enormous size. Big data samples frequently represent a substantial fraction of the entire population [155, 156]. This process trades off exactness and stability with completeness and consistency of the proxy observations. Thus, in BHD studies, the classical notion of statistical significance morphs into scientific inference that is based on joint modeling of all elements of big data using exploratory, classification, and pattern-tracking methods. Other essential distinctions exist between standard statistical analysis methods and advanced data analytics techniques [157]. Computational efficiency, data management, validation and reproducibility need Big-Data-specific, agile and scalable algorithms and models to obtain reliable inference on complex and heterogeneous data. The heterogeneity [158], noise concentration [3], spurious correlations [159], incidental endogeneity (hidden correlations between data elements and error terms) [160], and variable latency [161] that characterize big data also demonstrate the major challenges associated with handling, modeling and information extraction of BHD.

Data heterogeneity reflects the unavoidable differences in population characteristics, data formatting and type variability [162]. Big data always include heterogeneous data elements where small sub-samples might capture specific cohorts that include outliers or extreme data. An important property of big data that makes them useful is the population coverage of the data, asymptotically with the increase of the sample size. This enables us to model, stratify, and understand the heterogeneity of multiple sub-cohorts in the population. At the same time, noise concentration may creep in due to the aggregation of heterogeneous data elements and the accumulation of individual error terms into the joint big data analysis. Developing predictive big data analytic models requires simultaneous estimation of multiple parameters, model coefficients or likelihoods. In this joint processing, error estimates might compile (noise aggregation can be linear or non-linear in terms of the number of variables) and thus dominate the variable effect sizes or obfuscate the true effect of a parameter included in the model.

Spurious effects refer to data elements that are not associated in reality but that, owing to data complexity, are falsely determined to be significantly correlated [163]. For example, correlation coefficients between independent random variables can increase with the increase of the data size, incongruences in noise levels or the presence of latent variable effects. Another important factor in all Big Data analytic studies is the ‘curse of dimensionality’, which arises in dealing with high-dimensional data. This paradox is not present in traditional low-dimensional datasets. In high-dimensions many numerical analyses, data sampling protocols, combinatorial inferences, machine learning methods, or data managing processes are susceptible to the ‘curse of dimensionality’. Increases of data dimensionality (including a larger number of data elements) leads to parallel, and faster, increases of the space volume containing the observed data, thus, the actual points of data into the high-dimensional space appear to be drifting apart (distances between data points increases). The sparsity between points, even for big data, affects all quantitative analytic methods, as the corresponding statistical inference depends explicitly on the stability of ‘distance’ metrics [164]. The reliability of the statistical inference relies on balancing the volume of data (number of observation points) that needs to grow exponentially with the number of dimensions in which the data are embedded. In a high-dimensional space, objects may appear to be farther apart and artificially dissimilar, which affects data structuring, organization, modeling and inference. However, in big data studies, this problem of increased dimensionality and the associated challenges of interpreting data from multiple sources trades off with the potential for reduced bias, increased level of unique and heterogeneous population characteristics captured and broader interpretation of results.

Incidental endogeneity is a property that violates the common regression technique assumption that requires the independent (explanatory) variables to be independent of the error term (model residuals) [159]. Many parametric statistical methods depend on this assumption, as presence of incidental endogeneity allows potentially strong dependences between some predictors and the residuals that render the techniques possibly unreliable or underpowered. In traditional studies involving standard datasets the exogeneity assumption is usually met, that is, no acute incidental endogeneities occur. However, in BHD analyses, the expectation is that incidental endogeneity may be ubiquitous [165]. A difference exists between spurious effects and incidental endogeneity: the former refers to pseudo-random relationships, whereas the latter refers to natural intrinsic associations between the explanatory variables and the model residual error term.

### Data harmonization and fusion

When interpreting the information content of large and heterogeneous data, the processes of extraction of patterns, trends and associations demand considerable insights, computational power and analytical tools. Raw and derived data might come from multiple unrelated sources, and latent effects or multivariate correlations might complicate data interrogation. Traditional databases have bottlenecks in ingesting, retrieving and processing vast amounts of heterogeneous data. Modern structured query language (SQL) and NoSQL databases [166, 167], platforms for extract-transform-load processing [168] and cloud-based services [169–171] are improving the human and machine interfaces to BHD. Incongruent data often arrive from disparate digital sources, which can represent orthogonal, co-linear, or causally related information. Solid foundation for analytical and computational representation of big data is important. Alternative data representation schemes, canonical models or reference frameworks that facilitate data harmonization and integration across different granularity scales, encoding protocols, measurement types, phenotypes and formats are being developed [172, 173]. In practice, data incongruity can be due to the lack of such a common data representation architecture. Incompatibility of data elements is ubiquitous and unavoidable in most studies of real health data that rely on data-driven inference or evidence-based decision-making. Variable transformations, data imputations, low-dimensional modeling, and joint analyses all depend on a common scheme for effective representation of complex BHD. The implicit data harmonization necessary to enable subsequent data integration and processing is predicated on successful wrangling and fusion of incongruous data elements.

### Services and infrastructure

The MapReduce model from Google provides an attractive mechanism for parallel processing and *ad hoc* inference for large and heterogeneous datasets [174, 175]. A pair of functions, a mapper and a reducer, split real-world computational tasks (e.g. data cleaning, modeling, machine learning, filtering, aggregation, merging, etc.) into manageable scalable pieces that can be independently completed in parallel using separate parts of the (Big) data. These tasks could be performed on separate (but connected) machines even under failing node conditions. Hadoop is an open-source implementation of MapReduce [176]. The open-source Apache http://spark.apache.org/ enables distributed computing for large and complex datasets. Spark and MapReduce are linearly scalable and fault-tolerant; however, Spark can be up to 100 times faster for certain applications and provides rich and intuitive machine interfaces (e.g. application program interfaces in Python, Java, Scala and R) to support data abstraction and a wide spectrum of computing-intensive tasks, interactive queries, streaming, machine learning and graph processing.

PMML [177] is an XML-based language for describing, assembling and sharing predictive models learned within a data mining process that facilitates computational processing (machine-to-machine communication and distributed manipulation). DataMining-as-a-Service (DMaaS) [178], DecisionScience-as-a-Service (DSaaS) [179], Platform-as-a-Service (PaaS) [180], Infrastructure-as-a-Service (IaaS) [181] and Software-as-a-Service (SaaS) [182] are all examples of cloud-based data, protocol and infrastructure services enabling reliable, efficient and distributed data analytics. R packages [124, 147], KNIME [183], WEKA [184], RapidMiner [185] and Orange [186] include hundreds of powerful open-source algorithms and software tools for high-throughput machine learning, data mining, exploration, profiling, analytics and visualization.

**Fig. 5:**
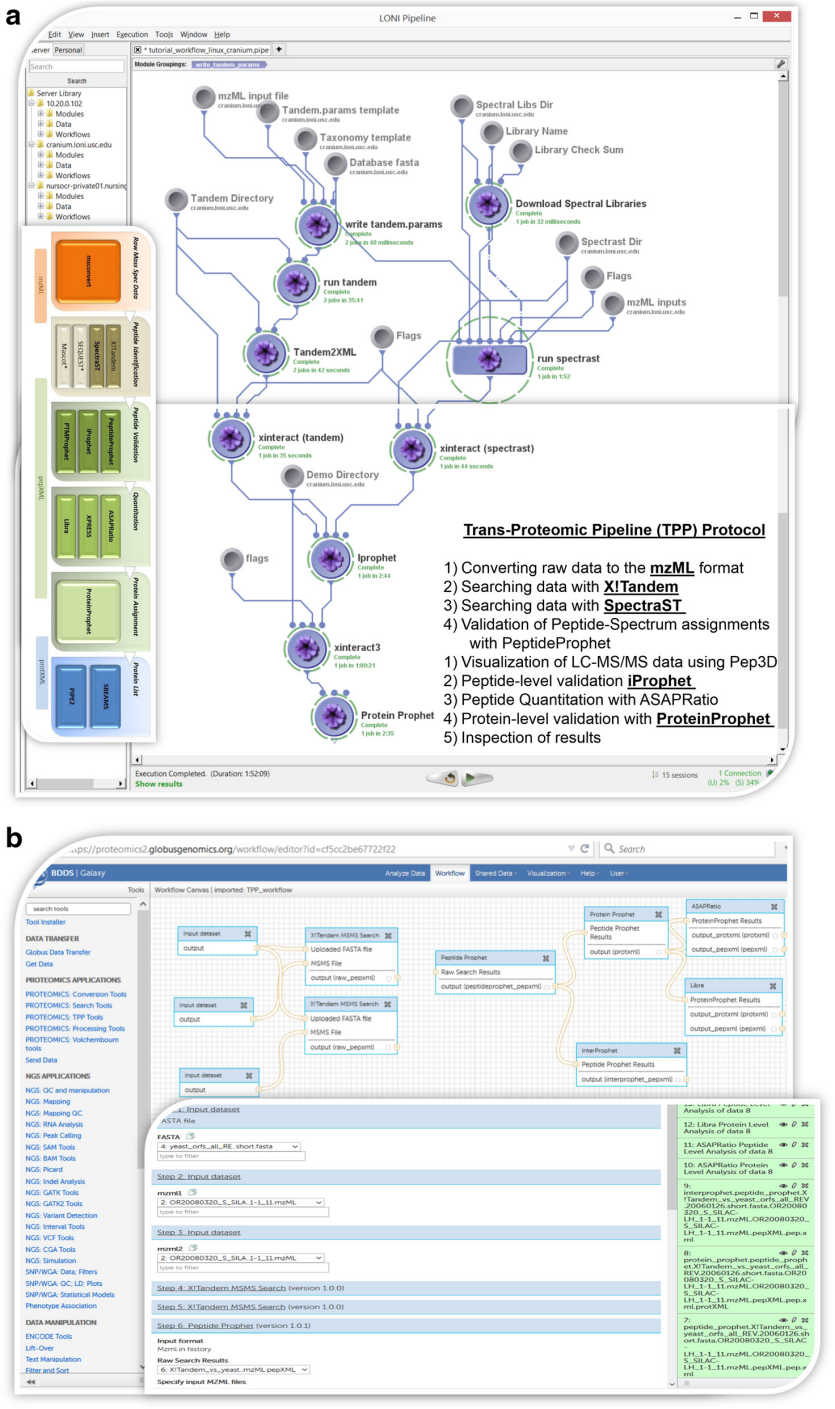
Two alternative end-to-end cloud-based solutions to the Trans-Proteomic Pipeline (TPP) protocol that apply advanced data modeling, processing and visualization methods to process mass spectroscopy datasets using multiple cloud web services. **a** Implementation of the TPP computational protocol in the Pipeline environment. **b** Implementation of the TPP computational protocol in the Globus Galaxies environment [205]

Figure 5 provides an example of a high-throughput end-to-end computational protocol in which several of such cloud web services are used. This example illustrates the implementation of the Institute for Systems Biology Trans-Proteomic Pipeline (TPP), which applies advanced data modeling, processing and visualization to the search and process datasets using multiple engines [187]. The dual Pipeline-based and Galaxy-based solutions are alternative service-oriented protocols that yield the same results using vastly different computational platforms. Many similar examples that use the Imaging Data Archive services [188, 189], Parkinson’s Progression Markers Initiative services [190, 191], Galaxy computational services [192], Pipeline client-server infrastructure [45, 193, 194] and proteomics services [195] are available online [196, 197].

Various national and international big data science initiatives have emerged as a response to sizeable financial support from government agencies, philanthropic organizations and industry partners to develop platforms enabling ‘open-science’, data sharing, collaborative development and transdisciplinary engagement. For example, in the USA, the National Institutes of Health funded 11 National big data to Knowledge Centers (BD2K) [198] and several satellite BD2K activities. In Europe, the Virtual Physiological Human initiative [199], the European Life-sciences Infrastructure for Biological Information [200] and the Translational Information & Knowledge Management Services [201] have secured resources to build and use open-source translational data, tools and services (e.g. tranSMART [202]) to tackle challenging problems.

## Conclusions

In the biomedical and healthcare community, managing, processing and understanding BHD pose substantial challenges that parallel enormous opportunities in understanding human conditions in health and disease, across location, time, and scale. Although no unique blueprint or perfect roadmap exist, the characteristics of the data, the underlying model assumptions, the computational infrastructure demands, and the application scope all have vital roles in the choices about how to guide, handle and analyze such complex data. The field of Big-Data-driven research discoveries bridges various scientific disciplines, advanced information and communication technologies, and multiple sources, and is rapidly evolving. We have outlined big data challenges, identified big data opportunities and presented modeling methods and software techniques for blending complex healthcare data and contemporary scientific approaches. We give examples of several techniques for processing heterogeneous datasets using cloud services, advanced automated and semi-automated techniques and protocols for open-science investigations. New technologies are still necessary to improve, scale and expedite the handling and processing of large data that are increasing in size and complexity [193]. At the same time, substantial methodological progress, powerful software tools and distributed service infrastructure are already in place to enable the design, simulation and productization of the future computational resources necessary to support the expected avalanche of data [203]. Big data analytics are likely to encounter some setbacks and some great advances in the next decade. Additional public, private and institutional investments in data acquisition, research and development, and computational infrastructure, along with education, will spur the involvement of bright young minds to tackle the huge big data challenges, reap the expected information benefits and assemble knowledge assets. Balancing proprietary, open-source and community commons developments will be essential for broad, reliable, sustainable and efficient development efforts. The influence of big data will go beyond financing, high-tech and biomedical research. Big data will be likely to touch every sector of the economy and their signature feature will be rapid on-demand team science.

## Abbreviations

BHD: big healthcare data
GMM: Gaussian mixture modeling
ICA: independent component analysis
PCA: principal component analysis
SVM: support vector machines
